# A Molar Pregnancy within the Fallopian Tube

**DOI:** 10.1155/2016/4367181

**Published:** 2016-12-04

**Authors:** Laura Allen, Charlotte Dawson, Patricia Nascu, Tyler Rouse

**Affiliations:** Department of Obstetrics and Gynaecology, Western University, London, ON, Canada

## Abstract

*Background*. Discussion of the incidence of molar pregnancy and ectopic pregnancy. Role of salpingostomy and special considerations for postoperative care.* Case*. The patient is a 29-year-old G7P4 who presented with vaginal bleeding in the first trimester and was initially thought to have a spontaneous abortion. Ultrasound was performed due to ongoing symptoms and an adnexal mass was noted. She underwent uncomplicated salpingostomy and was later found to have a partial molar ectopic pregnancy.* Conclusion*. This case illustrates the rare occurrence of a molar ectopic pregnancy. There was no indication of molar pregnancy preoperatively and this case highlights the importance of submitting and reviewing pathological specimens.

## 1. Introduction

Hydatidiform mole (HM) is a premalignant form of gestational trophoblastic disease that occurs from improper fetal and placental development [1, 2]. There are two types of HM: complete or partial, and these differentiate based on clinical presentation, chromosomal pattern, histology, and outcome [2, 3]. Partial HM, as with our case, occurs when the ovum is fertilized by either two sperm or one diploid sperm causing a triploid mole (69XXX, 69XXY, or 69XYY) [1, 3]. Partial HMs may be associated with a fetus, even allowing for a detection of fetal cardiac activity in some cases [4]. This, along with its rarity, can make ectopic HM a difficult diagnosis, consequently causing it to be overlooked for simple ectopic.

While ectopic pregnancy and molar pregnancy are not rare events (approximately twenty in every 1000 [5, 6] and one in every 500 to 1000 pregnancies [7], resp.), the combination of the two, an ectopic HM, is an extremely rare event. There have only been a small number of molar ectopic pregnancies reported in the literature with estimates of incidence being around 1.5 in every 1,000,000 pregnancies [8].

## 2. The Case

**Figure 1:**
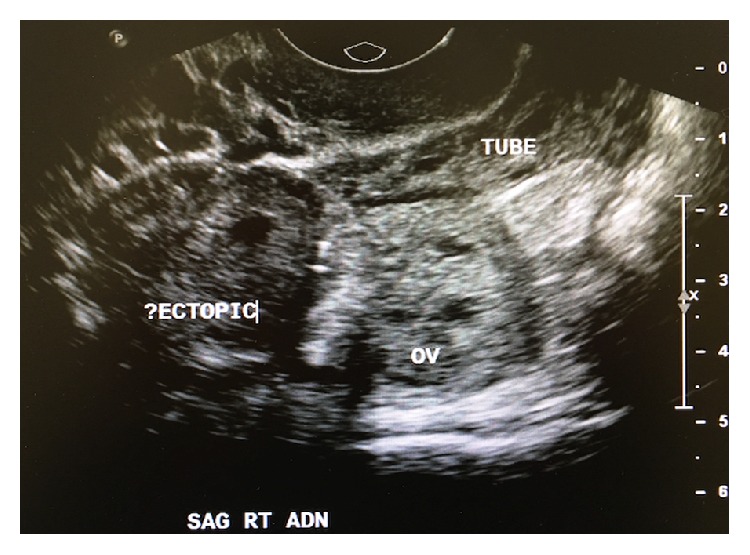
Transvaginal ultrasound of right adnexa.

The patient is a 29-year-old GTPAL 7, 4, 0, 2, 4 who presented with ongoing abdominal pain and ultrasound findings included a right sided mass in the adnexa measuring 2.2 by 2.4 by 2 cm with a fluid collection in the uterus measuring 4 by 0.4 by 2.2 cm with no evidence of a gestational sac (Figure 1). Laboratory findings include elevated serum beta human chorionic gonadotropin (BHCG) of 32000 IU/L. The remainder of her laboratory investigations was unremarkable.

She had been diagnosed with a spontaneous abortion a month previously after a positive pregnancy test and vaginal bleeding. Her past medical history was significant for obesity (BMI 30) and essential hypertension requiring no medications at present. Her past obstetrical history included a previous ectopic pregnancy, two previous spontaneous early trimester losses (including this recent supposed spontaneous abortion), two previous uncomplicated vaginal deliveries, and two previous Cesarean sections. Her past surgical history was remarkable for a left salpingectomy for an ectopic pregnancy, umbilical hernia repair, appendectomy, and cystoscopy.

The patient presented to the emergency room with complaints of abdominal pain. She was hemodynamically stable, in no distress with an unremarkable physical exam. Given the findings of an adnexal mass and no evidence of a gestational sac despite sufficiently elevated BHCG the most likely diagnosis is an ectopic pregnancy. The patient consented to laparoscopy, right salpingostomy, possible right salpingectomy, and dilation and curettage (D&C).

Laparoscopic entry was uncomplicated and a mass was visualized in the right fallopian tube. There was some dark blood pooling in the cul de sac, but no obvious tubal rupture. Salpingostomy was performed using monopolar cautery and the ectopic pregnancy was removed from the ampulla in an Endo Catch bag. Rh immunoglobulin was given postoperatively and she had an uncomplicated course following the surgery.

**Figure 2:**
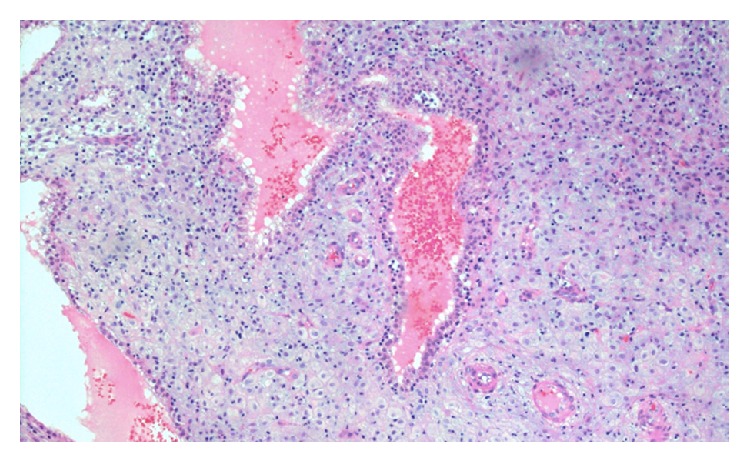
Fallopian tube with decidual change.

**Figure 3:**
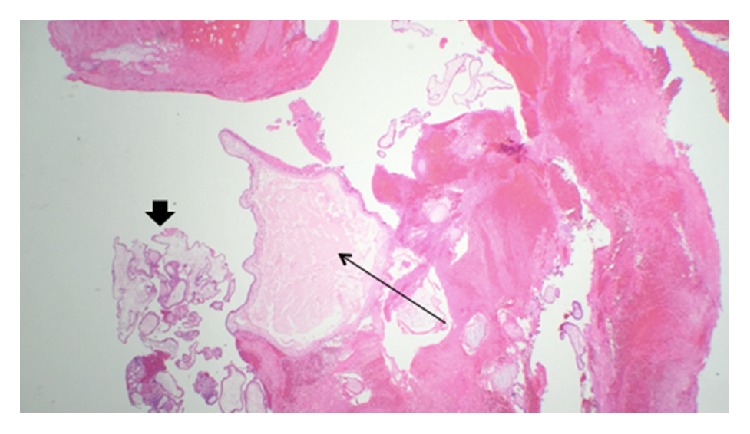
Low power magnification (2x) of the partial hydatidiform mole. Thin arrow: large edematous villus with central cistern formation. Thick arrow: smaller villi.

The final pathology revealed atypical chorionic villi (Figures 2 and 3) in keeping with a partial hydatidiform molar pregnancy in the right fallopian tube and decidualized endometrium was noted in the D&C specimen.

Given these findings the patient was observed closely with serial bloodwork monitoring BHCG level; BHCG was zero one month following surgery. It has been two years since her surgery and there is no evidence of persistent disease. The patient was seen in clinic one year later for fertility counseling and the patient did not return following that appointment.

## 3. Discussion

The patient in our case was at risk of recurrent ectopic pregnancy given an ectopic pregnancy in the past [9]. Risk factors for HM are not as clearly defined as for ectopic, but a history of multiple spontaneous abortions is gaining evidence as a factor in HM pregnancy [10]. Previous cases of ectopic molar often required salpingectomy for definitive treatment; here we were able to preserve her remaining fallopian tube.

The gold standard for diagnosis of HM is through histopathology [11–13]. Clinical presentation is usually indistinguishable from simple ectopic pregnancy [6], and diagnosis is usually made after laparoscopy or laparotomy upon pathological examination. Previous incidence of ectopic HM may be overestimated due to limited compliance with stringent histological criteria [11, 13, 14]. The diagnosis of HM may be confused by nonmolar hydrotropic villi changes seen in nonmolar ectopic pregnancies [15]. Careful consideration should be used in distinguishing the two, as HM has the potential to cause persistent trophoblastic disease and requires careful follow-up and monitoring [13]. The use of DNA flow cytometry has sometimes been used as a complement to histological diagnosis [11]. In the case of the patient in question, she had repeated BHCG levels performed until zero.

## 4. Conclusions

Ectopic molar pregnancy remains a very rare occurrence, thus making it an often overlooked diagnosis. Ectopic molar pregnancy should be considered in women presenting clinically with a suspected ectopic pregnancy, as ectopic HM will mimic the presentation. Diagnosis should be made through histopathology and augmented with DNA flow cytometry. Given how rare ectopic molar pregnancies are, there is no data about whether salpingostomy is a safe alternative to salpingectomy. Regardless all patients with an extrauterine pregnancy are followed to rule out persistent tissue.
